# Filariasis in Travelers Presenting to the GeoSentinel Surveillance Network

**DOI:** 10.1371/journal.pntd.0000088

**Published:** 2007-12-26

**Authors:** Ettie M. Lipner, Melissa A. Law, Elizabeth Barnett, Jay S. Keystone, Frank von Sonnenburg, Louis Loutan, D. Rebecca Prevots, Amy D. Klion, Thomas B. Nutman, for the GeoSentinel Surveillance Network

**Affiliations:** 1 Office of Global Research, National Institute of Allergy and Infectious Diseases, National Institutes of Health, Bethesda, Maryland, United States of America; 2 Laboratory of Parasitic Diseases, National Institute of Allergy and Infectious Diseases, National Institutes of Health, Bethesda, Maryland, United States of America; 3 Division of Travel and International Health, Boston University Medical Center, Boston, Massachusetts, United States of America; 4 Division of Infectious Diseases, University of Toronto, Toronto, Canada; 5 Department of Tropical and Infectious Diseases, University of Munich, Munich, Germany; 6 Travel and Migration Medicine Unit, Department of Community Medicine, Geneva University Hospital, Geneva, Switzerland; Leiden University Medical Center, The Netherlands

## Abstract

**Background:**

As international travel increases, there is rising exposure to many pathogens not traditionally encountered in the resource-rich countries of the world. Filarial infections, a great problem throughout the tropics and subtropics, are relatively rare among travelers even to filaria-endemic regions of the world. The GeoSentinel Surveillance Network, a global network of medicine/travel clinics, was established in 1995 to detect morbidity trends among travelers.

**Principal Findings:**

We examined data from the GeoSentinel database to determine demographic and travel characteristics associated with filaria acquisition and to understand the differences in clinical presentation between nonendemic visitors and those born in filaria-endemic regions of the world. Filarial infections comprised 0.62% (*n* = 271) of all medical conditions reported to the GeoSentinel Network from travelers; 37% of patients were diagnosed with *Onchocerca volvulus*, 25% were infected with *Loa loa*, and another 25% were diagnosed with *Wuchereria bancrofti*. Most infections were reported from immigrants and from those immigrants returning to their county of origin (those visiting friends and relatives); the majority of filarial infections were acquired in sub-Saharan Africa. Among the patients who were natives of filaria-nonendemic regions, 70.6% acquired their filarial infection with exposure greater than 1 month. Moreover, nonendemic visitors to filaria-endemic regions were more likely to present to GeoSentinel sites with clinically symptomatic conditions compared with those who had lifelong exposure.

**Significance:**

Codifying the filarial infections presenting to the GeoSentinel Surveillance Network has provided insights into the clinical differences seen among filaria-infected expatriates and those from endemic regions and demonstrated that *O. volvul*us infection can be acquired with short-term travel.

## Author Summary

As international travel increases, there is rising exposure to many pathogens not traditionally encountered in the resource-rich countries of the world. The GeoSentinel Surveillance Network, a global network of medicine/travel clinics, was established in 1995 to detect morbidity trends among travelers. Filarial infections (parasitic worm infections that cause, among others, onchocerciasis [river blindness], lymphatic filariasis [e.g. elephantiasis, lymphedema, hydrocele] and loiasis [African eyeworm]) comprised 0.62% (*n* = 271) of the 43,722 medical conditions reported to the GeoSentinel Network between 1995 and 2004. Immigrants from filarial-endemic regions comprised the group most likely to have acquired a filarial infection; sub-Saharan Africa was the region of the world where the majority of filarial infections were acquired. Long-term travel (greater than 1 month) was more likely to be associated with acquisition of one of the filarial infections than shorter-term travel.

## Introduction

Parasitic diseases are widespread throughout the developing world and are associated with a heavy burden of morbidity and mortality. Human filariae, nematodes transmitted by arthropod vectors, are endemic in tropical and subtropical regions of the world. With an estimated 80 million people who travel to developing countries each year [1], exposure to filarial parasites is likely to become more common. It has been suggested that infection with filariae requires prolonged and intense exposure to the vectors that transmit them [2]. Moreover, when comparing nonendemic visitors who have acquired filarial infections with those born in endemic regions, the nonendemic visitors appear to have greater numbers of objective clinical symptoms and fewer clinically asymptomatic (or subclinical) infections [3]–[7].

The GeoSentinel Surveillance Network, a global network of specialized travel/tropical medicine clinics on six continents, was established in 1995 to contribute clinician-based sentinel surveillance on all travelers seen [8]. We examined data from the GeoSentinel database to identify demographic and travel characteristics associated with filaria acquisition in addition to species distribution of filarial acquisition and patient symptoms. Because there have been no comprehensive studies that have addressed the acquisition of filarial infections among nonendemic travelers, the present study was performed to understand travel-related filarial infections from a global viewpoint that could inform physicians and travelers alike.

## Methods

### Data source

Demographic, travel, and clinical data were collected from all patients seen at each GeoSentinel site. Travel information was also collected, including trip start and end dates for travel within 6 months and countries visited in the previous 5 years. Countries listed included birth country, country lived in prior to age 10, country of residence, and country of citizenship. Patient classification, the reason for recent travel, symptoms, and final diagnosis were reported by health care providers at GeoSentinel site clinics. Patient information was entered without identifiers into an Access database (Microsoft). Each individual record with a diagnosis of filarial infection was examined manually to verify that the place of exposure was in a filaria-endemic country and that the data provided were accurate and complete.

The GeoSentinel data-collection protocol was reviewed by the institutional review board officer at the National Center for Infectious Diseases at the Centers for Disease Control and Prevention and classified as public health surveillance and not as human-subjects research requiring submission to institutional review boards.

### Inclusion criteria

Data entered into the GeoSentinel database from patients seen from August 1997 through December 2004 were used. This analysis focused on data extracted from persons who were assigned codes corresponding to infection with *Onchocerca volvulus, Wuchereria bancrofti, Loa loa*, other filarial species, or unknown filarial species. Prior to analysis, a survey of all GeoSentinel sites was performed to ensure that the definition of infection was uniform among the reporting sites.

### Definitions and groupings

#### Patient classifications

Patients were classified into seven categories: immigrants/refugees, foreign visitor, urban expatriate, non-urban expatriate, student, traveler, military. These categories were based on country of origin, place of GeoSentinel site visit, and purpose of travel. An immigrant was defined as someone born and raised in a filarial-endemic region. A traveler was defined as one who crossed an international border and returned to his/her country of residence and presented to a clinic site. A foreign visitor was someone who sought medical care at a GeoSentinel site during their trip but was not a resident or citizen of that country. Persons who emigrated from one filaria-nonendemic country to another filaria-nonendemic country and classified as ‘immigrant’ were reclassified to an appropriate category. Students from filaria-endemic regions studying in nonendemic regions were reclassified from student to immigrant for the purposes of these analyses. Persons born and raised in filarial nonendemic regions and traveling to filarial-endemic regions are collectively referred to as “nonendemic visitors”.

#### Reason for recent travel

The reasons for recent travel were categorized into immigration, tourism, business, research/education, missionary/volunteer, or visiting friends or relatives (VFR) based on patient self-report to physician. VFR are people born and raised in a filaria-endemic region, but currently residing in a filarial nonendemic region. Students from filaria-endemic regions studying in nonendemic regions were reclassified from education to immigrant for the purposes of these analyses.

#### Diagnoses

Physician-reported final diagnoses were assigned a diagnosis code and entered into the GeoSentinel database. Diagnoses are defined as suspect, probable, or confirmed. Confirmed means that the diagnosis was made by an indisputable clinical finding or diagnostic test (identification of the parasite or parasite DNA), and probable indicates that the diagnosis was supported by evidence strong enough to establish presumption (classical clinical findings and positive serology, and response to definitive treatment), but not proof. All sites used the best available reference diagnostics in their own country. Some of the ‘filarial species unknown’ diagnoses were reclassified into *O. volvulus, W. bancrofti*, or *L. loa* if the country of exposure had only one filarial species present. Of the 65 originally classified as unknown filarial species, 50 were reclassified.

#### Regions

Countries were grouped into regions: Southeast Asia, Eastern Europe, Northern Africa (including Canary Islands), Oceania, Western Europe, sub-Saharan Africa, South America, Caribbean, South Central Asia (including Tibet), Western Asia, Australia/New Zealand, North America, Antarctica, Eastern/North Asia (including Taiwan), and Central America.

#### Duration of travel, and time to presentation to a GeoSentinel site

Detailed travel data were available for only a subset of patients. In those for whom this information was available, trip duration and time to presentation were divided into 1 month, 1-6 months, and over 6 months to group short, medium, and long-term exposure and incubation periods. Those without definitive travel data related to the place of exposure were excluded only from this particular type of analysis. Trip duration and time to presentation were determined only for those who did not have lifelong exposure to filarial infections. The time to presentation was the interval between the clinic visit and date of return from the most recent travel to a filarial-endemic region of the world.

### Statistical analysis

Data were managed in Microsoft Access and were analyzed using SAS v.9.1 (SAS Institute). Crude odds ratios were calculated from a bivariate analysis, and statistical significance was determined by χ^2^ tests.

## Results

**Figure 1:**
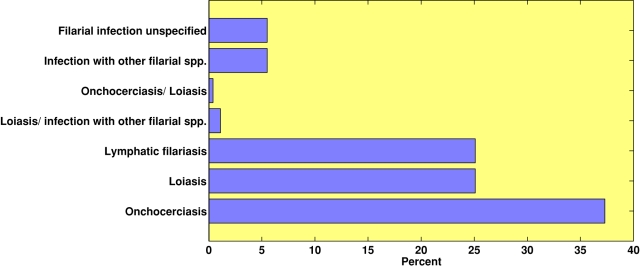
Distribution of filarial infections among international travelers reported in the GeoSentinel Surveillance Network.

From a total of 43,722 individual patient encounters, filarial infections were diagnosed for 271 (0.62%) persons who presented to GeoSentinel sites from August 1997 through July 2004. The reporting of cases to GeoSentinel was lowest in 1997 and 1998 (3.7% and 8.9% respectively); from 1999 through 2004, filariasis as a proportion of morbidity (ill patients reporting to the clinics) fluctuated between 11% (*n* = 30) and 17.5% (*n* = 47). Of the 271 patients with filarial infections, 37% were diagnosed with *O. volvulus*, 25% were infected with *L. loa*, and another 25% were diagnosed with *W. bancrofti*. Among all filarial infections, 5.5% were identified as other filarial species, (*e.g*., Mansonella, Brugia spp.), and 5.5% of all filarial infections reported in the database were unspecified. Three patients were coinfected with *L. loa* and other filarial species; one patient presented with *O. volvulus* and *L. loa* coinfection (Figure 1). Overall, 122 (45%) patients were female; gender was not recorded for 17 (6.3%) patients. Patient mean age was 34.9 years (range 0–84). The region of acquisition among filaria-infected individuals was assigned when possible (*n* = 230). The majority (75%) of infections were acquired in Africa (both Northern Africa and Sub-Saharan Africa) and 10% in South America (see Table 1). The remaining individuals were exposed in, Oceania, the Caribbean, South Central Asia, and Central America. Of all filarial infections reported to the GeoSentinel ntwork (*n* = 271), the majority were reported by the North American sites (76.4%); 18.5% were reported from European sites, and the remainder were reported from GeoSentinel sites in the Middle East, Australia/New Zealand, and South Central Asia.

**Table 1:** Region and countries of exposure to filarial parasite

Region	Country	N (%)
Africa		172 (75.1%)
	Benin	2
	Burkina Faso	3
	Burundi	1
	Cameroon	62
	Central African Republic	6
	Comoros	1
	Congo	5
	Cote d'Ivoire	3
	Egypt	1
	Ethiopia	9
	Gabon	7
	Ghana	9
	Guinea	2
	Liberia	11
	Nigeria	10
	Senegal	1
	Sierra Leone	10
	Tanzania	1
	Niger	2
	Sudan	4
	Togo	1
	Uganda	1
	Unspecified	18
	Zaire	2
South America		23 (10%)
	Brazil	1
	Guyana	22
South Central Asia		15 (6.6%)
	Bangladesh	1
	India	8
	Nepal	1
	Sri Lanka	5
Caribbean		8 (3.5%)
	Dominican Republic	2
	Haiti	6
		
South East Asia		5 (2.2%)
	Philippines	3
	Vietnam	2
Oceania		4 (1.7%)
	Guam	1
	Papua New Guinea	1
	Samoa	1
	South Pacific Islands	1
Central America		2 (0.9%)
	Mexico	1
	Nicaragua	1
Total		229 (100%)

**Figure 2:**
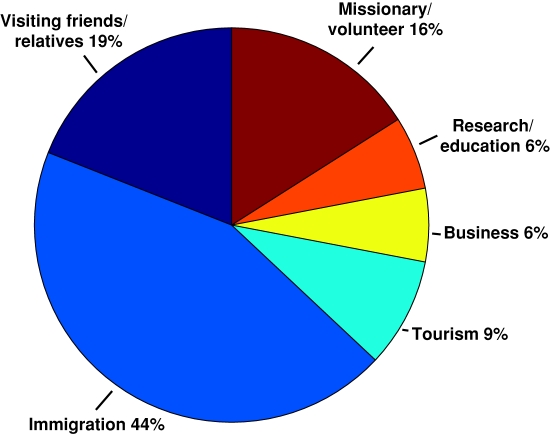
Patient classification among persons with filarial infections reported in the GeoSentinel Surveillance Network.

**Figure 3:**
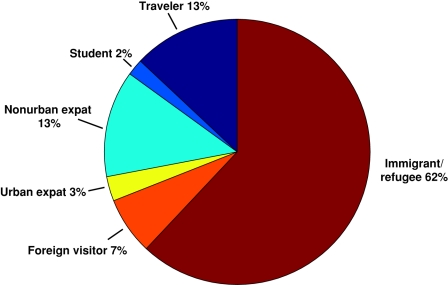
Reason for travel among persons with filarial infections reported in the GeoSentinel Surveillance Network.

**Figure 4:**
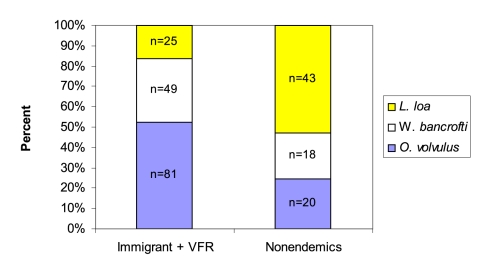
Regional distribution of filarial infections among immigrants, VFR, and nonendemic visitors reported in the GeoSentinel Surveillance Network.

Among the 271 patients diagnosed with filarial infections, the majority (62%) occurred among immigrants. Non-urban expatriates and travelers represented the second largest group of patients with filarial infections. Foreign visitors, urban expatriates, and students (Figure 2) comprised the groups in which there were relatively few filarial infections. As an overall proportion of GeoSentinel reports, filarial infections were found to occur in 1.6% of immigrants, 2.4% of non-urban expatriates, 1.5% of students, 0.2% of foreign visitors, 0.2% of urban expatriates, and 0.2% of travelers. The ‘reasons for travel’ were predominantly for immigration or for immigrants who were VFR in endemic regions (63%). An additional 16% of patients traveled for missionary or volunteer activities, and the remainder traveled for tourism, research/education, or business-related purposes (Figure 3). When grouped by type of parasite, immigrants and VFR had the greatest proportion of diagnosed onchocerciasis (48%) compared with nonendemic visitors (20%). Twenty-nine percent of VFR and immigrants with filarial infections were infected with *W. bancrofti*, while only 18% of nonendemic visitors had *W. bancrofti* infection. The diagnosis of *L. loa* was greatest among nonendemic visitors (43%), compared with 15% of VFR and immigrants with loiasis (Figure 4).

Travel duration was known definitively for 108 of the 271 individuals with filarial infection. Among these 108, 48 persons originated from nonendemic regions but only 34 had recorded travel data definitively related to the place of exposure. Trip duration ranged from 7 days to 17.7 years (geometric mean duration: 125 days; median duration: 87 days). The majority of patients with *O. volvulus* infections had trip durations of up to 1 month (Table 2). The majority of those with *L. loa* infections had traveled between 1 and 6 months, while the highest percentage of patients with *W. bancrofti* infection occurred after more than 6 months of travel (and presumed exposure).

**Table 2:** Trip duration by filarial infection among nonendemic visitors

# Days	*O. volvulus n* (%)	*W. bancrofti n* (%)	*L. loa n* (%)	Other filarial spp. *n* (%)
0–31	7 (77.8)	0	2 (12.5)	1 (14.3)
32–180	1 (11.1)	0	9 (56.3)	1 (14.3)
>180	1 (11.1)	2 (100)	5 (31.2)	5 (71.4)

The time to presentation to a GeoSentinel site after arrival in a filaria-nonendemic country was calculated to identify the possible incubation period between exposure and clinical presentation in only nonendemic visitors (VFR and immigrants excluded from this analysis). For *O. volvulus* infections, 67% presented to a GeoSentinel site within 1 month of return, and 100% of those with *W. bancrofti* presented between 1 and 6 months. Among those with *L. loa*, 12% presented within the first month of return, 77% within 1 to 6 months of return, and the remainder at least 6 months after return. Among patients infected with other filarial species, the majority presented within 1 month of return. These data suggest that Onchocerca infections are more likely to be symptomatic early in the infection compared to either *Loa loa* or *Wuchereria bancrofti* (data not shown).

In studies done previously in loiasis [3] and onchocerciasis [4] among limited numbers of expatriates, the data suggested that the clinical symptoms were more pronounced (and less likely to be asymptomatic) in travelers (temporary visitors) to filaria-endemic regions of the world compared with those with lifelong exposure and chronic infections [3]. To examine this issue more closely, a comparison was made between those infections that were clinically symptomatic and those that were clinically asymptomatic (Table 3). Characterization of symptoms included those associated with the following organ systems: skin, cardiac, respiratory, gastrointestinal, genitourinary, neurologic, musculoskeletal, ophthalmologic, and otolaryngologic, in addition to complaints of fatigue, fever, and psychological problems. If the patient had no complaints or symptoms or in which filarial infection was identified incidentally following evaluation for another condition, then asymptomatic was recorded. As seen, those individuals in the GeoSentinel database identified to have filarial infection who were born and raised in endemic regions were 2.5 times as likely to be clinically asymptomatic (CI 1.07, –5.93) compared with those who traveled from filaria-nonendemic to filaria-endemic regions (*P*<.03)

**Table 3:** Association between patient endemicity status and commonly presenting symptoms of filarial infections

Symptomatic	Endemic	Nonendemic	Total
No	35	7	42
Yes	152	75	227
Total	187	82	269
		Missing: 2	
OR (95% CI)	2.5 (1.05, 5.81)		

## Discussion

While filarial infection and disease are most frequently diagnosed among native residents of endemic regions, the risk of infection acquisition among travelers from nonendemic regions is sizeable. Filarial species are found in tropical and sub-tropical regions of the world and, as travel to these regions becomes more popular, filarial infection among nonendemic visitors becomes increasingly common as well. We describe here important epidemiologic characteristics of filarial infections acquired by world travelers from nonendemic regions as reported to the GeoSentinel network. While clinical presentation of filarial disease is known to differ between visitors to and natives of endemic regions [3], our analysis also provides a quantitative assessment of filarial acquisition among travelers and helps describe the differences in clinical presentation between those native to filaria-endemic regions and those traveling to those regions.

Filarial infections comprised 271 cases (0.62%) of all medical conditions reported to the GeoSentinel network. *O. volvulus* was responsible for the greatest number of filarial infections (*n* = 101), followed by equal numbers (*n* = 68) of *L. loa* and *W. bancrofti* (Figure 1). Because the GeoSentinel database includes immigrants/refugees who undergo laboratory screening that includes filarial serologies when eosinophilia or clinical signs or symptoms of filarial disease are present, it is not surprising that the majority of filaria-infected patients in the GeoSentinel network were immigrants (62%). Due to lifelong chronic exposure, the prevalence of filarial infections among immigrants can be significant.

It has, however, typically been said that infection acquisition is low for short-term, nonendemic travelers. Although travel information was only available for a subset of the total number of filaria-nonendemic visitors, it was still unexpected to find that almost one-third (30%) of travelers from nonendemic regions acquired their filarial infections during trips of 31 days or less (the majority of *O. volvulus* infections), and only 38% of filarial infections occurred from trip durations exceeding 180 days (Table 2). There are numerous case reports and case series that describe durations of exposure as short as 10 days among filaria-infected patients from nonendemic regions [5], [9]–[12]. It is possible that the lack of preventive measures such as insect repellent and bednets, as well as individuals close proximity to vector habitats, played a role in infection acquisition regardless of short durations of exposure. Further, development of symptoms may also be dependent on the density of filarial larval inoculation as well as individual innate immune responses [13].

Because almost all of the major filarial infections (*O. volvulus, W. bancrofti, L. loa, M. perstans, M. streptocerca*) are endemic in sub-Saharan Africa, it is not surprising that 72% of filarial infections reported to GeoSentinel were acquired in this region: 95.5% of those with onchocerciasis were acquired in sub-Saharan Africa; three were acquired elsewhere. Thirty-two percent of the *W. bancrofti* infections were acquired in South America, compared with only 12% *o*f *W. bancrofti* infections reported from sub-Saharan African regions, 22% from South Central Asia, and 14% from the Caribbean. As expected, 100% of loiasis cases were acquired in West and Central Africa, as the parasite is endemic only in this region.

While short-term nonendemic visitors appear less likely to acquire filarial infections, among those with relatively long-term exposure there have been many case reports of travel-related filarial infections and associated clinical symptoms [3]–[5], [11], [13]–[15]. Presentation of clinical disease among patients with *L. loa, O. volvulus*, and *W. bancrofti* differs considerably between expatriates (or long-term temporary residents) and those born in filaria-endemic regions of the world. Among those infected with *L. loa*, infected expatriates typically have a greater frequency of Calabar swellings, higher grade levels of filaria-specific antibody and peripheral eosinophil counts, and more nonspecific complaints, while those born and raised in endemic regions are more likely to have asymptomatic infections associated with microfilaremia. Those born in regions with endemic *O. volvulus* infection generally have higher levels of skin microfilariae and more ocular disease than do nonendemic visitors to these regions [16]. Those living in regions with endemic lymphatic filariasis most commonly have asymptomatic (or subclinical) infections, although significant proportions of infected individuals develop hydrocele, lymphedema elephantiasis, or chyluria. Nonendemic visitors (and short-term visitors) rarely have asymptomatic microfilaremic condition, but rather are more likely to develop lymphadenitis, hepatomegaly, splenomegaly and reversible lymphedema [17].

This study corroborates many of the anecdotal reports about the differences between the clinical presentations among travelers compared with those with chronic (and often lifelong) exposure to filarial parasites. Case report findings describe the clinical manifestations of filarial disease to be greater among expatriates, while those from filaria-endemic regions present commonly without symptoms. Indeed, our results from the GeoSentinel network indicate that filaria-infected patients with long-term exposure to filariae were more commonly asymptomatic (or subclinical) compared with those expatriates with filarial infections.

With the collection of surveillance data on travel-related medical conditions by the GeoSentinel network, epidemiologic data can describe morbidity and mortality trends among travelers [18]. While these networks are generally used to follow acute infections among nonendemic visitors, we have demonstrated here the utility of surveillance for chronic infections, as well. Diagnoses of filarial infections in industrialized countries will likely continue to rise as increasing numbers of people travel to endemic regions and as increasing numbers of refugees and immigrants arrive from endemic areas. The majority of nonendemic filaria-infected visitors (64.7%) presented to a GeoSentinel site clinic between 1 and 6 months after return of travel, underscoring the need for surveillance of chronic infections to ensure safety and treatment of returning travelers from developing regions.

In conclusion, analysis of data on filarial infections available from the GeoSentinel network enabled us to describe characteristics of patients presenting with filarial infection and to determine that filarial infections can be acquired with relatively short-term exposure. Our study not only corroborates but expands the understanding of the differences in filarial disease manifestation between those traveling to and those born in filaria-endemic regions of the world by providing a quantitative analysis of filarial acquisition among nonendemic visitors. Moreover, our data demonstrate that globally acquired travel data can be used to follow not only acute but also chronic infections and can ultimately provide a more comprehensive backdrop to pre-travel advice and to post-travel treatment for those at risk of acquiring a filarial infection.

## Members of the Geosentinel Surveillance Network

In addition to the authors, members contributing data include:

Graham Brown and Joseph Torresi, Royal Melbourne Hospital, Melbourne, Australia; Giampiero Carosi and Francesco Castelli, University of Brescia, Brescia, Italy; Lin Chen, Mount Auburn Hospital, Harvard University, Cambridge, Massachusetts, USA; Bradley Connor, Cornell University, New York, New York, USA; Jean Delmont and Philippe Parola, Hôpital Nord, Marseille, France; Carlos Franco and Phyllis Kozarsky, Emory University, Atlanta, Georgia, USA; David Freedman, University of Alabama, Birmingham, Alabama, USA; Stefanie Gelman and Devon Hale, University of Utah, Salt Lake City, Utah, USA; Alejandra Gurtman, Mount Sinai Medical Center, New York City, New York, USA; Jean Haulman and Elaine Jong, University of Washington, Seattle, Washington, USA; Kevin Kain, University of Toronto, Toronto, Canada; Carmelo Licitra, Orlando Regional Health Center, Orlando, Florida, USA; Prativa Pandey, CIWEC Clinic Travel Medicine Center, Kathmandu, Nepal; Patricia Schlagenhauf and Robert Steffen, University of Zurich, Zurich, Switzerland; Eli Schwartz, Sheba Medical Center, Tel Hashomer, Israel; Marc Shaw, Travellers Health and Vaccination Centre, Auckland, New Zealand; Mary Wilson, Harvard University, Cambridge, Massachusetts, USA; Murray Wittner, Albert Einstein School of Medicine, Bronx, New York, USA.
